# Next-generation sequencing-based molecular diagnosis of neonatal hypotonia in Chinese Population

**DOI:** 10.1038/srep29088

**Published:** 2016-06-29

**Authors:** Yan Wang, Wei Peng, Hong-Yan Guo, Hui Li, Jie Tian, Yu-Jing Shi, Xiao Yang, Yao Yang, Wan-Qiao Zhang, Xin Liu, Guan-Nan Liu, Tao Deng, Yi-Min Sun, Wan-li Xing, Jing Cheng, Zhi-Chun Feng

**Affiliations:** 1 BaYi Children’s Hospital, Beijing Military General Hospital, Beijing, 100700, P.R. China; 2 National Engineering Research Center for Beijing Biochip Technology, Beijing, 102206, P.R. China; 3 CapitalBio Corporation, Beijing, 102206, P.R. China; 4 Beijing CapitalBio Medical Laboratory, Beijing, 101111, P.R. China; 5 Department of Biomedical Engineering, Tsinghua University School of Medicine, Beijing, 100084, P.R. China

## Abstract

Neonatal hypotonia is extremely challenging to diagnose because numerous disorders present similar clinical manifestations. Two panels for diagnosing neonatal hypotonia were developed, which enriches 35 genes corresponding to 61 neonatal hypotonia-related disorders. A cohort of 214 neonates with hypotonia was recruited from 2012 to 2014 in China for this study. Of these subjects, twenty-eight neonates with hypotonia were eliminated according to exclusion criteria and 97 were confirmed using traditional detection methods. The clinical diagnoses of the remaining 89 neonates with hypotonia were approached by targeted next-generation sequencing (NGS). Among the 89 tested neonates, 25 potentially pathogenic variants in nine genes (*RYR1, MECP2, MUT, CDKL5, MPZ, PMM2, MTM1, LAMA2* and *DMPK*) were identified in 22 patients. Six of these pathogenic variants were novel. Of the 186 neonates with hypotonia, we identified the genetic causes for 117 neonates by the traditional detection methods and targeted NGS, achieving a high solving rate of 62.9%. In addition, we found seven neonates with RETT syndrome carrying five mutations, thus expanding the mutation profiles in Chinese neonates with hypotonia. Our study highlights the utility of comprehensive molecular genetic testing, which provides the advantage of speed and diagnostic specificity without invasive procedures.

Neonatal hypotonia is a nonspecific clinical sign frequently associated with central or peripheral nervous system abnormalities, myopathies, endocrinopathies, metabolic diseases and acute or chronic illnesses. The clinical manifestations of hypotonic neonates are soft limbs and weak sucking ability, which leads to feeding difficulties. Neonatal hypotonia is common, but challenging to diagnose because numerous disorders present similar clinical manifestations. The proportion of genetic diseases reported in studies of neonatal hypotonia ranges from 19.2% to 60.0%^1^,^2^,^3^,^4^, due to the limitations of testing methods, different ethnic groups and sample sizes. At present, a variety of diseases and genes have been associated with neonatal hypotonia^5^,^6^, demonstrating the extreme genetic heterogeneity of these diseases.

Accurate molecular diagnosis and genetic mechanism studies are essential for treatment, prognosis, genetic counseling and prenatal diagnosis of neonatal hypotonia. To date, the molecular diagnostic tools for this disease include Sanger sequencing, PCR-based methods and next-generation sequencing. Sanger sequencing is considered the gold standard for mutation screening, but is costly and time-consuming for large-scale sequencing. PCR-based methods are only applicable to single diseases, such as spinal muscular atrophy (SMA) and Prader-Willi syndrome^7^,^8^,^9^,^10^,^11^, leading to a low diagnosis rate. NGS is the newest, most efficient technology for the screening and detection of genetic variation. One approach of NGS is targeted sequencing, in which only the functional regions of the interested genes for a specific disease or diagnostic category are amplified^12^. Targeted NGS has been widely used in the molecular diagnosis of pediatric diseases such as monogenic diabetes^13^, Duchenne muscular dystrophy (DMD)^14^ and mucolipidosis II alpha/beta^15^,^16^,^17^. Targeted NGS provides a superior quality of representation, and simplifies the resulting dataset analysis, as well as the interpretation of the variants compared with whole exome sequencing (WES) or whole genome sequencing (WGS)^18^. These qualities make targeted NGS the optimal strategy for molecular diagnosis of neonatal hypotonia.

Many valuable reviews and studies have been published addressing the clinical and genetic evaluation of neonatal hypotonia, but they did not have a specific description of the molecular diagnostic strategy, and considered it only from a theoretical point of view^5^,^6^,^19^,^20^. The establishment of new molecular diagnostic methods and the diagnostic profile of neonatal hypotonia will improve our treatment strategy. In this study, we applied a targeted NGS-based sequencing system for neonates with hypotonia in China. Our primary objective was to improve diagnostic efficiency by developing a panel of known genes associated with neonatal hypotonia using targeted NGS. The secondary objective was to gain insight into the diagnostic profile of the different diseases that can cause neonatal hypotonia in a large retrospective study of the Chinese population.

## Results

### Characteristics of subjects with neonatal hypotonia

**Figure 1:**
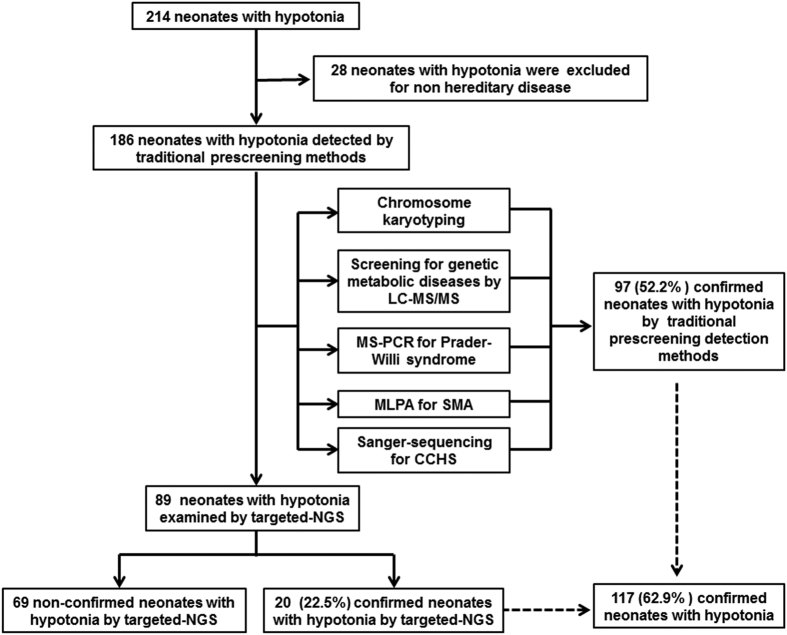
Workflow diagram of the molecular genetic diagnosis. During the period from January 2012 to December 2014, a total of 214 neonates with a predominant problem of hypotonia were recruited. Among the 214 neonates with hypotonia, 28 were eliminated for non-hereditary disease. By traditional prescreening detection methods, 97 (52.2%) were confirmed to have genetic diseases associated with hypotonia. Further targeted NGS reassessment resulted in a refinement of the clinical diagnosis in 20 patients. In total, genetic causes were identified for 117 of 186 neonates with hypotonia, achieving a solving rate of approximately 62.9%.

A total of 214 neonates with major symptoms of hypotonia were recruited in this study. Of these candidates, 28 were eliminated according to the exclusion criteria (Table S1). We therefore selected the remaining 186 neonates with hypotonia for the subsequent analyses. Of the 186 neonates with hypotonia, 97 (52.2%) were confirmed (central in 87 neonates with hypotonia and peripheral in 10 neonates with hypotonia) on the basis of the traditional detection methods. The clinical etiology of the remaining 89 prescreened neonates with hypotonia was undetermined and thus further examined by targeted NGS (Fig. 1). Among the 186 neonates with hypotonia (Table 1), 65.1% (n = 121) were boys. The mean age was 8.5 days on admission to the hospital (SD = 6.4 days) and seven of the babies were less than three days old. 55.9% (n = 104) of the neonates with hypotonia had swallowing difficulties, and 46.7% (n = 87) had respiratory distress symptoms.

### Targeted NGS of 89 patients

The genomic DNAs derived from the 89 neonates with hypotonia were sequenced. All of them were subjected to the capture panel, whereas 54 samples were conducted with both capture and amplicon panels. Additionally, the genomic DNAs of 13 healthy people were sequenced as negative controls with both panels.

**Figure 2:**
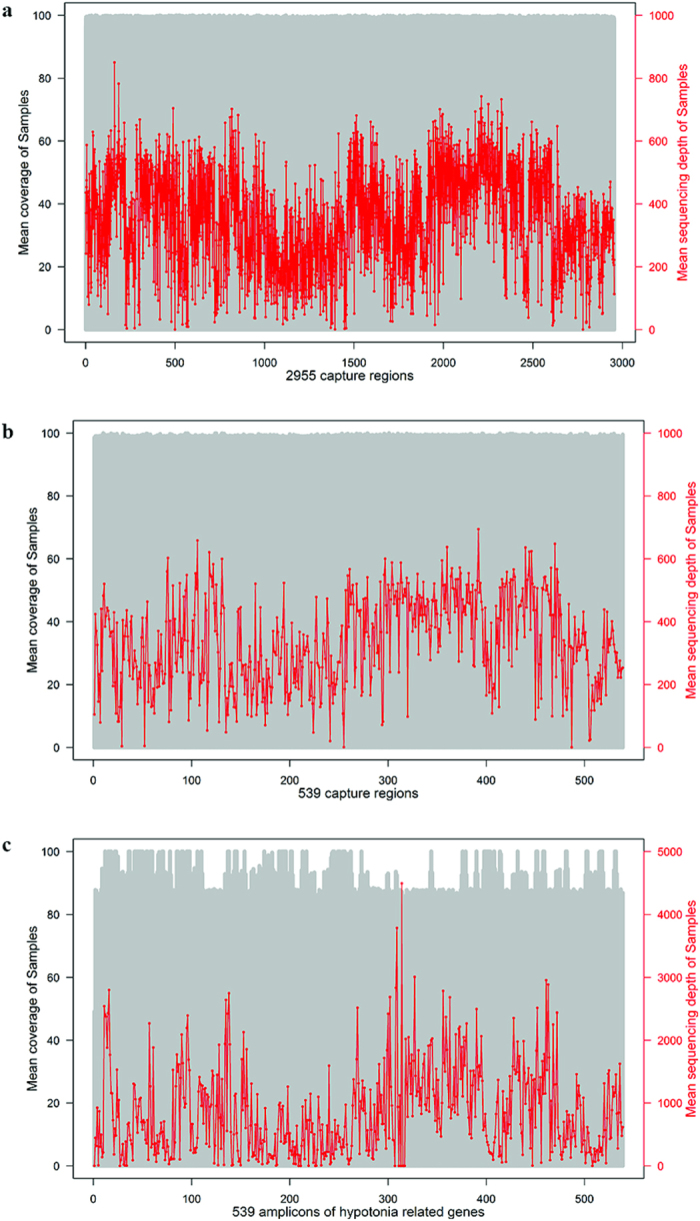
Distribution of coverage depth for targeted regions. (**a**) The mean coverage depth of 2955 capture regions covering four kinds of monogenic disorders. (**b**) The mean coverage depth of 539 capture regions of hypotonia-related genes. (**c**) The mean coverage depth of 539 amplicons of hypotonia-related genes. The grey histogram represents the mean coverage in each region. The red line represents the mean sequencing depth of each region.

A capture panel was designed to enrich the target DNA consisting of 953,749 bp, which covers 35 neonatal hypotonia associated genes (Table S2) and 169 genes of other monogenic diseases. The total target DNA encompassed 2,955 capture regions covering four kinds of monogenic disorders, and 539 of those capture regions were associated with the 35 hypotonic genes. The DNAs from 89 samples were sequenced with 265~1,542 Mb per sample. The mean depth was 175~983×, with the mean coverage of all of the targeted regions being 95.2~99.8%. For the 35 neonatal hypotonia associated genes, the mean depth was 168~931× with the mean coverage of 95.2~99.5%. In addition, 54 samples were subjected to amplicon sequencing. The cumulative target, which includes 539 amplicons, was approximately 112 kb. For these samples, the amount of data was 147~266 Mb per sample. On average, the mean coverage depth was 643~1154× with the mean coverage of 91.5~93.7% of the targeted regions. The mean depth and coverage of the targeted regions by both NGS panels were further analyzed and showed in Fig 2, both the coverage and the uniformity of the capture sequencing is better than those of amplicon sequencing.

### Mutation identification

To identify the potential mutations among the rare variants identified in our cohort of neonates with hypotonia by the NGS panels, we checked the variants for matching inheritance patterns of respective genes and their affected phenotypes. In this study, we reported four potential pathogenic mutations of the 35 genes, which include the following: (1) homozygous or compound heterozygous variants in recessive disease-causing genes; (2) heterozygous variants known to cause dominant diseases; (3) heterozygous loss of function (LOF) variants including stop gain, splicing, and frame shift variants newly reported in dominant disease genes, and homozygous or compound heterozygous LOF variants newly reported in recessive disease genes; (4) heterozygous missense variants newly reported to be pathogenic in dominant disease genes, and missense homozygous or compound heterozygous variants newly reported to be pathogenic in recessive disease genes. This is in accordance with *in silico* prediction with KGGSeq software^21^.

**Figure 3:**
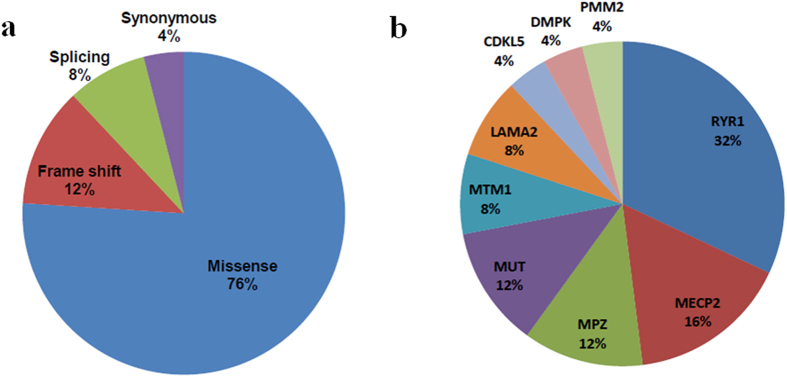
The targeted next generation sequencing statistics for potentially pathogenic mutations. (**a**) The distribution of different types of pathogenic mutations identified in 22 patients. (**b**) The distribution of pathogenic mutations in different genes.

A total of 25 potentially pathogenic mutations were identified in the 22 of the 89 hypotonia neonates by using the Roche NimbleGen capture sequencing platform whereas the 19 potentially pathogenic mutations were identified in 16 of the 54 hypotonia neonates by using the illumina TruSeq amplicon sequencing method (Table S3). The potentially pathogenic mutations identified in the samples that have been sequenced by both targeted NGS methods were completely identical, meaning that the data were highly credible. These 25 mutations included 19 missense (76.0%), 1 synonymous (4.0%), 2 splicing (8.0%), and 3 frameshift mutations (12.0%) (Fig. 3a). Notably, 6 of these pathogenic mutations, representing about 24.0% of total mutations, were identified as novel mutations (Table 2). These 25 potentially pathogenic mutations belong to nine genes (Fig. 3b). The most prevalently mutated gene was *RYR1*, which explained Central core/Multi-minicore disease/King-Denborough syndrome in six of the neonates, and included eight mutations. The second most observed gene was *MECP2*, in which defects can lead to RETT syndrome, and in which four mutations were flagged in six participants (Table 2). All those candidate mutations were not determined in the healthy control samples and were confirmed by Sanger sequencing, which showed the consistency between the NGS and Sanger sequencing.

### Reported genotype–phenotype correlation

Our study identified ten heterozygous variants in genes, *RYR1*, *DMPK*, *LAMA2*, *MECP2* and *MPZ*, which were also previously reported to be uncertain or undetermined significance in the dbSNP database. These ten variants were predicted to be pathogenic with KGGSeq (http://statgenpro.psychiatry.hku.hk/limx/kggseq/) (Table 2). Among these ten variants, five were located in the *RYR1* with one splicing and four missense mutaions. It has been known that dominant and recessive mutations in *RYR1* could cause a range of muscle disorders, though many other aspects of disease pathogenesis involving this gene remain uncertain. Two missense mutations in *LAMA2* gene of patient 135 (c.2217G>T & c.4640C>T) were compound heterozygous. Congenital myodystrophy caused by the *LAMA2* mutations showed an autosomal recessive (AR) inheritance pattern. Meanwhile, two heterozygous mutations on *RYR1* gene (c.5746G>C & c.8417G>A) were also detected, on patients 58 and 175, respectively. Patient 143 carried a heterozygous missense mutation in the *MECP2* gene. Further analysis is required to establish whether the heterozygous state can cause a mild phenotype. In our cases, the minor allele frequency of these mutations was less than 1% in the 1000 genome project and NHLBI GO Exome Sequencing Project database (https://esp.gs.washington.edu).

Ten carried mutations were reported in the primary literature and exhibited corresponding phenotypes. Missense mutation c.602C>T (p.Ala201Val) in gene *MECP2* was the most frequent mutation and carried by three girls. For patients 194, 187 and 137, they had different hemizygous mutations in different genes, which caused Rett syndrome. Rett syndrome is an X-linked dominant (XD) disease. Patient 162 carried compound heterozygous reported missense mutation c.1280G>A (p.Gly427Asp) and frameshift insertion c.729_730insTT (p.Asp244LeufsX39) in *MUT*. Patient 213 had heterozygous reported missense mutation c.389A>G (p.Lys130Arg) in *MPZ*, which caused Charcot-Marie-Tooth disease with dominant inheritance model. Two patients (No. 216 and 217) were carrier with only one heterozygous missense mutation for related diseases with recessive inheritance model.

### Novel genotype–phenotype correlation

Five patients carried 6 novel mutations that predicted to be pathogenic. Among these five patients, two of them (No. 219 and 202) carried LOF mutations that severely affected protein functions, and the others carried one or two missense mutations that were predicted to have deleterious protein function effects *in silico*. Patient 219 carried a homozygous mutation in *MTM1* (c.231+2T>C), which likely lead to RNA mis-splicing. The mutation of *MTM1* is known to show a phenotype of myotubular myopathy, with inheritance of X-linked recessive. People with X-linked myotubular myopathy have muscle weakness (myopathy) and decreased muscle tone (hypotonia) that are usually evident at birth^22^. Patient 202 carried one heterozygous frameshift mutation (c.632delA), and an existing variant (c.77C>T) without clinical significance in *MPZ*. The mutation of *MPZ* is reported to cause Charcot-Marie-Tooth disease, which appears to be an autosomal dominant genetic disease. Mutation of c.632delA changed the corresponding protein sequence after 210 amino acids and came into a truncated protein (p.Lys211SerfsX41), while the other mutation resulted Pro to Leu at 26 amino acid sites. Compared to missense mutation, the frame shift mutation is more likely responsible for the disease.

Two patients were detected with compound heterozygous mutations, with both having *RYR1* mutations. All of the *RYR1*-related diseases have been reported with either AR or autosomal dominant (AD) inheritance models. Patient 120 carried compound heterozygous existing splicing (c.425-1G>A) and novel missense mutation (c.6982G>A) in the *RYR1* gene, and patient 232 carried compound heterozygous missense mutations (c.658C>T & c.4715T>C) in the same gene. The putative mutations of patient 232 were found co-segregation in her parents among the study cohort. Analysis of her normal parents revealed that the mother had the c.4715T>C missense mutation, while her father had the c.658C>T missense mutation. Patient 252 carried a hemizygous novel missense mutation in the *MTM1* gene. The identification of novel genotype–phenotype correlations enlarged the mutation spectrum of neonatal hypotonia phenotypic and genotypic heterogeneity.

### The clinical diagnoses of neonatal hypotonia

The statistic result of clinical diagnoses of the neonates was listed in Table 3. Of the 186 neonates with hypotonia, we identified the genetic causes for 117 (62.9%) using both the traditional detection methods and NGS. Among these, the final causes for neonatal hypotonia were classified as central hypotonia in 95 (81.2%) neonates and peripheral in 22 (18.8%) neonates. The central hypotonia disorders identified fell into three separate clusters: chromosomal abnormalities/syndromic disorders (n = 77), metabolic/endocrinal disorders (n = 17) and other syndromes (congenital central hypoventilation syndrome) (n = 1). RETT syndrome was identified in seven neonates with hypotonia. The peripheral hypotonia disorders identified include Spinal muscular atrophy (n = 10), central core/multi-minicore disease (n = 6), Charcot-Marie-Tooth disease (n = 2), myotonic dystrophy (n = 1) and myotubular myopathy (n = 2).

## Discussion

In the past decade, a variety of diseases and associated genes have been linked to neonatal hypotonia^1^,^4^,^23^,^24^. The significant genetic heterogeneity of the disease categories is a great challenge for clinical molecular diagnosis. Traditional detection methods for individual genes are time consuming and can only provide limited mutation spectrum of the disease. NGS, which has revolutionized the molecular genetic research, is a high-throughput method capable of rapidly sequencing a large number of genes in parallel and providing large data sets^25^,^26^,^27^. In this study, we developed a capture NGS-based method for the molecular diagnosis of neonatal hypotonia. The capture panel covered 35 genes corresponding to 61 neonatal hypotonia-related disorders plus some other monogenic diseases. We systematically assessed this panel on the control samples and then applied it to 89 prescreened hypotonic neonates with unknown genetic variance. To exclude the bias of the targeted NGS platform, we have also developed an amplicon NGS-based method and tested 54 of the 89 hypotonic neonates. The potentially pathogenic mutations identified by both methods were identical, proving the technology was highly credible. Additionally, 20 patients were diagnosed by the NGS screening and phenotype confirmation, which represents approximately 22.5% unknown neonates with hypotonia. By a combination strategy of traditional prescreening methods and subsequent NGS panel screening, 62.9% of neonates with hypotonia were confirmed economically and efficiently. To the best of our knowledge, this is the first study that investigated the application and validation of a targeted NGS-based method in neonatal hypotonia, and our sample cohort presents the largest group of unrelated neonates that has been screened for known neonatal hypotonia genes.

One of the important findings presented here is the 6 novel mutations, which represent 24.0% of the determined mutations in our cohort. These rare variants may have greater impact on disease pathogenesis compared with the more common variants^28^,^29^,^30^. With the completion of the 1000 genome project, the field of human population genetics is entering an era in which rare variants are systematically characterized^31^. Therefore, a significant number of novel mutations will be discovered by NGS-based molecular diagnosis, and a high diagnosis rate will be achieved. Among the 6 novel mutations identified in our study, two were LOF mutations and the others were missense mutations (Table 2). All of these novel mutations might be potential pathogenic variants. Firstly, none of these mutations were found in RefSeq, dbSNP, ClinVar and CapitalBio GEDD database. Secondly, the pathogenic potential of all of the novel missense mutations was supported by KGGSeq using a logit model to combine prediction scores from nine multiple methods. Thirdly, the mutations reported in our study are consistent with the genetic model of the genes associated with disease. Besides, the affected cases were consistent with the reported phenotypes. Novel pathogenic mutation candidates have now been added to the known mutations of the neonatal hypotonia genes. These mutations can build a resource for understanding the genetic contribution to the diseases responsible for the condition^32^.

In our study, 25 mutations were identified from 22 genetic defects including two carriers. Therefore, the positive detection rate by NGS was 22.5%. Our patient cohort had been prescreened by the traditional technology, such as karyotyping, LC-MS/MS, MS-PCR, MLPA and Sanger sequencing, and then detected by NGS. In total we identified the genetic causes for 117 of 186 hypotonic neonates, achieving a high solving rate of approximately 62.9%. Two main factors can explain the other 37.1% remaining participating newborns, for which we could not determine the causative mutations. First, some infrequent or novel disease-causing genes were not included in our panel. Second, these undetermined cases may be due to mutations that were not covered by our method, including gene recombination, large structure variations, copy number variations and epigenetic variations. These unsolved cases should be valuable to identify novel disease-causing genes by using the WES or WGS technology.

To our knowledge, this is the first report of the diagnostic disease spectrum in Chinese neonates with hypotonia. In our study, the diagnostic disease spectrum in Chinese neonates with hypotonia was diverse. Our results confirm that central causes of neonatal hypotonia are more common than peripheral causes, in a nearly 5.3:1 ratio (Table 3), which is consistent with the proportion of approximately 80.0% central causes found in other studies^1^,^4^,^33^. In our cohort, a chromosomal abnormality disorder (Trisomy 21) accounts for 25.3%, metabolic and endocrinal disorders are present in 9.1%, syndromic disorders, including Prader-Willi syndrome, make up 12.4% and RETT syndrome was found in 3.8%. The highest proportion of cases in our peripheral hypotonia group comprises cases of SMA and CMD^23^,^33^. In an analysis from five clinical series, chromosomal abnormalities accounted for 0–29.7%, syndromic disorders were shown in 4.9–21.7%, metabolic disorders in 0–9.6%^1^,^2^,^3^,^4^,^33^. The profiles and contribution of the other genetic causes of the hypotonia complex in our cohort are different from those identified in other studies (Table 4).

Interestingly, seven neonates with RETT syndrome were found in our study, which is different from other studies^1^,^2^,^3^,^4^,^33^(Table S4). RETT syndrome is usually identified later, and hypotonia is not a presenting sign during the neonatal period. RETT syndrome typically appears from 6–18 months, followed by regression of development. Other manifestations for RETT syndrome are hypotonia, autism, ataxia and stereotypical hand movements. Mutations in the gene *MECP2* encoding the X-linked methyl-CpG-binding protein 2 were detected in 80–90% of patients with typical RETT syndrome, and in only 40% patients with atypical RETT syndrome^34^. Mutations in the *CDKL5, MEF2C, FOXG1* and *TCF4* genes have also been reported in some cases of RETT syndrome^35^. In our study, among the seven neonates with hypotonia under the category of RETT syndrome, six carried mutations in the *MECP2* gene, and the other exhibited a homozygous missense mutation, c.216T>A in *CDKL5*. Among the six neonates with hypotonia who carried mutations in the *MECP2* gene, three carried the same heterozygous missense mutation, c.602C>T, and exhibited atypical phenotype of hypotonia and/or difficult feeding. At present, few reports of hypotonic neonates with RETT syndrome have been published, and only Heilstedt *et al*.^24^ reported that a girl, six months old, with a previously described mutation in the *MECP2* gene presented with hypotonia and developmental delay in infancy without a clear period of normal development. Our results support broadening the phenotype of patients who could be considered for mutation analysis of genes associated with RETT syndrome to include cases of hypotonia, when there is no evidence of an initial period of normal development. This is in agreement with Heilstedt and co-worker’s study.

In summary, we have developed a targeted sequencing methodology for the molecular diagnosis of neonates with hypotonia and identified pathogenic mutations for 25.0% of this prescreened patient cohort. A total of 6 novel pathogenic mutations were found. Furthermore, we found seven neonates with RETT syndrome and expanded the mutation profiles of disease in Chinese neonates with hypotonia. The identification of novel genotype-phenotype correlations and the mutation spectrum not only greatly enhances the heterogeneity knowledge of neonatal hypotonia, but also assists in both the clinical diagnosis and the development of personalized treatments. Our study highlights the utility of comprehensive molecular genetic testing, which provides the advantage of speed and diagnostic specificity without invasive procedures.

## Materials and Methods

### Study subjects

A cohort of neonates with predominant hypotonia phenotypes were recruited between January 2012 and December 2014 from the neonatal intensive care unit at the BaYi Children’s Hospital at the General Military Hospital of Beijing PLA. Subjects were only included if hypotonia had been first noticed before the 28th day of life and lasted for at least two weeks. The following exclusion criteria were used: (1) less than 35 weeks of gestation age (because pathological hypotonia might not be reliably assessed for more premature neonates); (2) obvious non-hereditary disease (mainly including congenital heart failure, hypoxic-ischemic encephalopathy, intracranial hemorrhage, atelencephalia, lateral ventricle cysts, intracranial infection and recurrent intra-spinal canal placeholders). By checking the medical records or by interviewing the participants’ guardians, we found that all cases and controls were genetically unrelated Han Chinese. This study was performed with the approval of the Ethical Committee of the General Hospital of Beijing PLA and was conducted according to the principles expressed in the Declaration of Helsinki. Written consent was obtained from all of the patients’ guardians.

### DNA extraction

Genomic DNA was extracted from peripheral blood leukocytes using the QIAamp DNA Blood Mini Kit (Qiagen, Valencia, CA) following the manufacturer’s instructions. The DNA purity (OD260/280 and OD260/280 ratio) were measured by a NanoDrop ND-1000 spectrophotometer (NanoDrop Technologies, Wilmington, DE). The DNA quality and integrity were assessed by the electrophoresis on a 1.0% agarose gel. The DNA quantification was performed by using the Quant-iT Broad-Range dsDNA Assay Kit and a Qubit 2.0 Fluorometer (Both from Thermo Fisher, Waltham, MA).

### Traditional prescreening detection methods

Chromosome karyotyping was performed on conventional GTG-banded metaphase cells^34^ prepared from the neonates who met the inclusion criteria. Screening for genetic metabolic diseases in neonates with hypotonia was performed with liquid chromatography-tandem mass spectrometry (LC-MS/MS)^35^. Methylation -specific polymerase chain reaction (MS-PCR) was used to diagnose Prader-Willi syndrome^36^. The multiple ligation-dependent probe amplification (MLPA) approach was used for the molecular diagnosis of SMA^37^. Sanger sequencing was used to detect congenital central hypoventilation syndrome (CCHS)^38^. The above methods were used as the first line molecular diagnostic methods when the patients met the inclusion criteria.

### Design of targeted enrichment panels

The accumulations of the knowledge of the clinical evaluation and investigation of neonatal hypotonia help us enlarge the diagnostic profile^1^,^2^,^4^,^5^,^6^,^20^,^33^. Further, some valuable studies^2^,^6^,^20^ have been published addressing the contributions of the relative genes in confirming the diagnosis. Sixty-one neonatal hypotonia-related disorders were selected from these literatures. Genes associated with these diseases were accessed from Genetic Home Reference (https://ghr.nlm.nih.gov/), which was included into custom panels for our retrospective study (Table S2). We developed two panels to enrich targeted DNA, capture panel and amplicon. For capture panel, 204 genes were selected corresponding to four types of monogenic diseases, including neonatal hypotonia, inborn errors of metabolism, children renal diseases and congenital immunodeficiency diseases. Of which, 35 genes have been reported with 61 neonatal hypotonia- related disorders. The amplicon panel only covered 35 known neonatal hypotonia-related genes. The mRNA accession numbers and related chromosome coordinates of the genes were downloaded from UCSC database (http://genome.ucsc.edu/cgi-bin/hgTables).

The probes for capture panel were designed according to NimbleDesign Software User’s Guide also using the H. sapiens hg19 primary assembly. The chromosome coordinates of coding exons, splicing sites and UTR were submitted to NimbleDesign (https://design.nimblegen.com/nimbledesign/app/login?execution=e3s1). The web-based Illumina Design Studio software was used to design custom amplicon oligonucleotide probes pool using the H. sapiens hg19 primary assembly. Parameters were adjusted in order to theoretically cover most of the exons and splicing sites, as well as all of the reported variants. Probes were synthesized by Illumina Inc. (San Diego, CA).

### Library preparation and sequencing

DNA was extracted from 89 prescreened neonates with hypotonia and 13 healthy controls. For capture procedure, the 250 ng of genomic DNA was fragmented into 200–300 bp by Covaris S220 system to construct DNA library. The library was generated and captured by using the NEBNext^®^ Ultra™ DNA Library Prep Kit for Illumina (NEB, Hitchin, Hertfordshire, UK) and the SeqCap EZ Choice Enrichment System (Roche NimbleGen, Madison, WI) according to the manufacturer’s instructions with minor modification respectively. Captured libraries were sequenced on the Illumina Hiseq 2500 sequencer as 100 bp paired-end read.

250 ng genomic DNA was used for each library preparation by using the TruSeq Custom Amplicon library Preparation Kit (Illumina). The Agencourt AMPure XP beads (Beckman Coulter, Beverly, MA) were used for PCR products clean-up. After library normalization, compatible indexed samples were pooled. Equal volumes of normalized libraries were combined, diluted in hybridization buffer, and denatured by heat before sequencing. The products were sequenced in multiplexed sequencing runs on the Illumina MiSeq Sequencer, generating 250 bp paired-end reads. Sequences were demultiplexed using MiSeq Reporter software, allowing 1 mismatch in the index sequence. Any reads that did not meet the overall quality as measured by the Illumina chastity filter were removed and the remaining reads were sorted by their index read sequence.

### Variant Detection and Annotation

For capture sequencing, the single nucleotide variation (SNV) were analyzed using the BWA (version 0.7.12)^39^ (http://bio-bwa.sourceforge.net/) and GATK programs (version 3.3)^26^ (https://www.broadinstitute.org/gatk/).The called variants were annotated with SNPEff (version 4.0)^40^ (http://sourceforge.net/projects/snpeff/), dbSNP Build 147^40^ (ftp://ftp.ncbi.nih.gov/snp/) and ClinVar database^41^ (http://www.ncbi.nlm.nih.gov/clinvar/) as well as CapitalBio GEDD database (CapitalBio, Beijing, China). The potentially pathogenic variants were evaluated with KGGSeq (version V0.5)^21^ using a logit model to combine prediction scores from multiple methods and to compute an unbiased estimate of the probability of a rare nonsynonymous SNV being pathogenic. Variants were filtered out with any of the following criteria to reduce false positive: (i) QUAL <50; (ii) MQ <20; (iii) GQ <40; (iv) DP <30; (v) refhomo_vf>0.05; (vi) het_vf <0.2; (vii) althomo_vf <0.75; (viii) MAF>0.01. For some cases, the het_vf between 0.15 and 0.2 were considered as pathogenic.

For amplicon sequencing, variants were called with MiSeq Reporter (Illumina) according to the user’s guide. In order to reduce false positive, variants were filtered out with any of the following criteria : (i) marked with“R8”; (ii) variant frequency <0.2; (iii) marked with“Low GQX”(iv) QUAL <50; (v) heterozygous SNP and Indel with depth of coverage <20; (vi) homozygous SNP with depth of coverage <4; (vii) homozygous Indel with depth of coverage <10; (viii) minor allele frequency >1%.

The final variants that passed the filters were re-annotated with Variant Effect Predictor Version 84^42^ (http://www.ensembl.org/Tools/VEP).

### Sanger sequencing validation

All of the putative mutations were validated by Sanger sequencing. The specific primers were designed by Primer3.0 online (http://primer3.ut.ee/). The PCR products were sequenced using BigDye Terminator v3.1 Cycle Sequencing Kit (Applied Biosystems, Foster City, CA) and analyzed by an ABI 3730 Genetic Analyzer (Applied BioSystems). The sequences were analyzed by the Mega6.0 (http://www.megasoftware.net/)^43^.

## Additional Information

**How to cite this article**: Wang, Y. *et al*. Next-generation sequencing-based molecular diagnosis of neonatal hypotonia in Chinese Population. *Sci. Rep*. **6**, 29088; doi: 10.1038/srep29088 (2016).

## Supplementary Material

Supplementary Information

## Figures and Tables

**Table 1:** Characteristics of the patient population.

	Total population(n = 186)	Central hypotonia(n = 87)	Peripheral hypotonia(n = 10)	Undetermined cases(n = 89)
Gender				
Boy	121	53	2	60
Girl	65	34	8	29
**Gestational age** (Mean), weeks	38.1	37.9	39.3	38.1
**Birth weight** (Mean), grams	2897	2843	3184	2918
**Birth weight** (SD), grams	567	545	543	586
Prenatal history				
Decreased fetal movements	20	8	4	8
Polyhydramnios	13	5	0	8
Intrauterine growth retardation	14	8	1	5
Mode of delivery				
Vaginal	57	29	3	25
Cesarean section	129	58	7	64
Syndrome				
Perinatal asphyxia	29	13	2	14
Respiratory distress	87	36	9	42
Feeding difficulties	104	55	9	40
Deaths	59	47	9	3

**Table 2:** Causative mutations and potentially pathogenic variants identified in this study.

Sample ID	Gene	Gender	Genotype	cDNA change	Protein change	Rs number	GMAF^a^	AA_MAF^b^	EA_MAF^c^	Disease presentation	Reference (PMID)^d^
Pathogenic prediction of the existing variants											
190	DMPK	M	Heterozygous	c.1790T>A	p.Val597Asp	rs201332435	T:0.0022	–	–	Myotonic dystrophy	7543316
135	LAMA2	M	Compound Heterozygous	c.2217G>T	p.Trp739Cys	rs192317605	T:0.0016	–	–	Merosin-deficient congenital muscular dystrophy type 1A	7550355
				c.4640C>T	p.Thr1547Met	rs778106503	–	–	–		
143	MECP2	F	Heterozygous	c.156C>G	p.His52Gln	rs781819534	–	–	–	Rett syndrome	17089071
202	MPZ	M	Heterozygous	c.77C>T	p.Pro26Leu	rs530923760	A:0.0002	–	–	Charcot-Marie-Tooth disease	8816708
58	RYR1	F	Heterozygous	c.5746G>C	p.Gly1916Arg	rs746965897	-	-	-	Central core disease; Multi-minicore disease; King-Denborough syndrome	12112081
											18765655
120	RYR1	M	Heterozygous	c.425-1G>A	Mis-splicing	rs745526344	–	–	–		
141	RYR1	M	Heterozygous	c.4113G>C	p.Arg1371Ser	rs551509462	C:0.0008	–	–		
175	RYR1	M	Heterozygous	c.8417G>A	p.Arg2806His	rs778214809	–	–	–		
179	RYR1	M	Heterozygous	c.14707G>A	p.Glu4903Lys	rs372418113	–	A:0	A:0.0001		
Ten patients carrying mutations reported in the primary literatures											
194	MECP2	M	Hemizygous	c.590C>T	p.Thr197Met	rs61749714	A:0.0003	A:0.0008	A:0.0003	Rett syndrome	12180070
61	MECP2	F	Heterozygous	c.602C>T	p.Ala201Val	rs61748381	A:0.0048	A:0.0003	A:0.0007		12180070
115	MECP2	F	Heterozygous	c.602C>T	p.Ala201Val	rs61748381	A:0.0048	A:0.0003	A:0.0007		12180070
207	MECP2	F	Heterozygous	c.602C>T	p.Ala201Val	rs61748381	A:0.0048	A:0.0003	A:0.0007		12180070
187	MECP2	M	Hemizygous	c.808delC	p.Arg270GlufsX19	rs62931162	–	–	–		10991688
162	MUT	M	Compound Heterozygous	c.1280G>A	p.Gly427Asp	rs753288303	–	–	–	Methylmalonicacidemia	16281286
				c.729_730insTT	p.Asp244LeufsX39	rs780283588	–	–	–		
217	MUT	M	Heterozygous	c.1208G>A	p.Arg403Gln	rs774457503	–	–	–	Methylmalonicacidemia	23430940
137	CDKL5	M	Hemizygous	c.216T>A	p.Ile72=	rs267608439	–	–	–	Rett syndrome	17089071
213	MPZ	M	Heterozygous	c.389A>G	p.Lys130Arg	rs281865127	–	–	–	Charcot-Marie-Tooth disease	8938258
216	PMM2	F	Heterozygous	c.422G>A	p.Arg141His	rs28936415	A:0.0036	–	–	Congenital disorder of glycosylation 1a	9140401
Five patients carrying novel mutations predicted to be pathogenic											
Patients carrying novel LOF mutations											
219	MTM1	M	Hemizygous	c.231+2T>C	Mis-splicing	–	–	–	–	Myotubular myopathy, X-linked	9450905
202	MPZ	M	Heterozygous	c.632delA	p.Lys211SerfsX41	-	-	-	-	Charcot-Marie-Tooth disease	8816708
Patients carrying one or more novel missense mutations											
120	RYR1	M	Heterozygous	c.6982G>A	p.Gly2328Arg	–	–	–	–	Central core disease; Multi-minicore disease; King-Denborough syndrome	12112081
232	RYR1	F	Compound Heterozygous	c.658C>T	p.Arg220Cys	–	–	–	–		18765655
				c.4715T>C	p.Met1572Thr	–	–	–	–		
252	MTM1	M	Hemizygous	c.1237A>C	p.Ser413Arg	–	–	–	**–**	Myotubular myopathy, X-linked	9450905

Gene and corresponding transcript: CDKL5:NM_003159.2; DMPK:NM_004409.3; LAMA2:NM_000426.3; MECP2:NM_004992.3; MPZ:NM_000530.6; MUT:NM_000255.3; MTM1:NM_000252.2; PMM2:NM_000303.2; RYR1:NM_000540.2. - indicates no information. Abbreviations: AD, autosomal dominant; AR, autosomal recessive; XD, X-linked dominant.

^a^GMAF: Non-reference allele and frequency of existing variant in 1000 Genomes.

^b^EA_MAF: Non-reference allele and frequency of existing variant in NHLBI-ESP European American population.

^c^AA_MAF: Non-reference allele and frequency of existing variant in NHLBI-ESP African American population.

^d^Reference (PMID): For mutations reported in primary literatures, references are these literatures including these mutations associated with related diseases. Otherwise, references are literatures that do not include these variants but illustrated the correlation between these genes and diseases, and the inheritance mode of these diseases. - indicates no information. Abbreviations: AD, autosomal dominant; AR, autosomal recessive; XD, X-linked dominant.

**Table 3:** Disease spectrums in Chinese neonates with hypotonia.

Diagnosis	Number of cases (%)
**Central hypotonia disorders**	95 (51.1)
Chromosomal abnormalities	
Down syndrome	47 (25.3)
Prader-Willis syndrome	23 (12.4)
Syndromic disorders	
RETT syndrome	7 (3.8)
Metabolic disorders	
Methylmalonic acidemia	11 (5.9)
Phenylketonuria	1 (0.5)
Propionic acidemia	1 (0.5)
Citrullinemia	1 (0.5)
Endocrinal disorders	
Hypothyroidism	3 (1.6)
Other syndrome	
Congenital central hypoventilation syndrome	1 (0.5)
**Peripheral hypotonia disorders**	22 (11.8)
Motoneuron disorders	
Spinal muscular atrophy	10 (5.4)
Congenital myopathies	
Central core/multi-minicore disease	6 (3.2)
Myotubular myopathy	2 (1.1)
Other muscle disorders	
Charcot-Marie-Tooth disease	2 (1.1)
Congenital myodystrophy	1 (0.5)
Myotonic dystrophy	1 (0.5)
**Undetermined**	69 (37.1)
**Total**	186

**Table 4:** Clinical case series of patients with hypotonia.

Clinical case	Total population	Central:peripheral	Central				Peripheral		Setting
			Chromosomal abnormalities	Syndromic disorders	Metabolic disorders	Endocrinal disorders	Spinal muscular atrophy	Congenital myopathies	
Richer *et al*.^2^	50	33:17(1.9)	4(8.0%)	9(18.0%)	0	0	1(2.0%)	7(14.0%)	Neonatal intensive care
Paro-Panjan and Neubauer^33^	138	121:13(9.3)	41(29.7%)	19(13.8%)	8(5.8%)	0	3(1.4%)	4(2.9%)	University Children’s Hospital
Vasta *et al*.^3^	83	44:39(1.1)	0	6(7.2%)	8(9.6%)	0	0	0	Neuromuscular unit
Birdi *et al*.^1^	60	58:12(4.8)	1(1.7%)	13(21.7%)	0	0	4(6.7%)	2(3.3%)	University Children’s Hospital
Laugel *et al*.^4^	144	98:22(4.5)	24(16.7%)	7(4.9%)	9(6.3%)	2(1.4%)	7(4.9%)	2(1.4%)	Tertiary care
Our research	214^a^	122:23(5.3)	47(22.0%)	30(14.0%)	14(6.5%)	3(1.4%)	10(4.7%)	8(3.7%)	Neonatal intensive care

^a^The 214 patients include 28 excluded by non-genetic diseases and 186 included in this study.
