# Supplementary material for: Next-generation sequencing-based molecular diagnosis of neonatal hypotonia in Chinese Population
Source: Sci Rep. 2016 Jun 29;6:29088. doi: 10.1038/srep29088 (PMC4926250; doi:10.1038/srep29088)
Supplement: Supplementary Information [file srep29088-s1.pdf]

Title page

Next-generation sequencing-based molecular diagnosis of neonatal hypotonia in Chinese Population

Yan Wang[1, #],Wei peng[1,#], Hong-Yan Guo[3,4] , Hui Li[3,4] , Jie Tian[3,4] , Yu-Jing Shi[3,4] , Xiao Yang[1], Yao Yang[1], Wan-Qiao Zhang[1], Xin Liu[1], Guan-Nan Liu[3,4] , Tao Deng[5], Yi-min Sun[3,4], Wan-li Xing[2,3,4], Jing Cheng[2,3,4 *], Zhi-Chun Feng[1,*]

Running title: Next-generation sequencing and neonatal hypotonia

Affiliations:

[1] BaYi Children's Hospital, Beijing Military General Hospital, Beijing, 100700, P.R. China

[2]Department of Biomedical Engineering, Tsinghua University School of Medicine, Beijing, 100084, P.R. China

[3] National Engineering Research Center for Beijing Biochip Technology, Beijing, 102206, P.R. China

[4] CapitalBio Corporation, Beijing, 102206, P.R. China

[5] Beijing CapitalBio Medical Laboratory, Beijing, 101111, P.R. China

[#] **These authors contributed equally to the paper.**

[*] **Correspondence:**

Zhi-Chun Feng

BaYi Children's Hospital, Beijing Military General Hospital, Beijing, 100700, P. R. China

Tel (+86)10-66721786; Fax (+86)10-64063099; E-mail: zhichunfeng81@163.com.

Jing Cheng

Department of Biomedical Engineering, Tsinghua University School of Medicine, Beijing, 100084, P.R. China.

Tel: (+86)10-62772239; Fax: (+86)10-62773059; E-mail: jcheng@tsinghua.edu.cn.

Table S1. The information of diseases for 28 excluded patients

Diagnosis	Number of cases
Hypoxic-ischemic encephalopathy	16
Intracranial hemorrhage	8
Atelencephalia	1
Lateral ventricle cysts	1
Intracranial infection	1
Recurrent intra-spinal canal placeholders	1
Total	28

Table S2. Panel of genes involved in neonatal hypotonia

**Disease association**	***Gene***	***Entrez Gene ID***	***Chromosome***	***Exon No.***	**Inheritance**
**Central hypotonia**					
Rett syndrome	*MECP2*	4204	chrX	4	XD
	CDKL5	6792	chrX	20	XD
Methylmalonic academia (include 7 subtypes)	*MUT*	4594	chr6	12	AR
	MMAA	166785	chr4	6	
	MMAB	326625	chr12	9	
	MCEE	84693	chr2	3	
	MMADHC	27249	chr2	7	
Peroxisomal disorders (include 21 subtypes)	*PEX1*	5189	chr7	24	AR
	PEX2	5828	chr8	1	
	PEX3	8504	chr6	12	
	PEX5	5830	chr12	17	
	PEX6	5190	chr6	17	
	PEX10	5192	chr1	7	
	PEX12	5193	chr17	3	
	PEX13	5194	chr2	4	
	PEX14	5195	chr1	9	
	PEX16	9409	chr11	12	
	PEX19	5824	chr1	9	
	PEX26	55670	chr22	5	
Congenital disorder of glycosylation (CDG)	*PMM2*	5373	chr16	8	AR
**Peripheral hypotonia**					
Myotonic dystrophy	*DMPK*	1760	chr19	19	AD
Spinal muscularatrophy (include 4 subtypes)	*SMN1*	6606	chr5	16	AR
Barth syndrome	*TAZ*	6901	chrX	11	XR
Charcot-Marie-Tooth disease(CMT) (include 6 subtypes)	*MPZ*	4359	chr1	6	AD
	PMP22	5376	chr17	4	
	EGR2	1959	chr10	3	
Myotubular myopathy	*MTM1*	4534	chrX	14	XR
Neuropathy, recurrent, with pressure palsies	*PMP22*	5376	chr17	4	AD
Neuropathy, congenital hypomyelinating, 1	*EGR2*	1959	chr10	3	AD, AR
Central core disease<br>Multi-minicore disease<br>King-Denborough syndrome	*RYR1*	6261	chr19	106	AR, AD

Congenital myodystrophy	Merosin-deficient congenital muscular dystrophy type 1A (MDC1A)	*LAMA2*	3908	chr6	65	AR
	Walker-Warburg syndrome (WWS) Muscular dystrophy-dystroglycanopathy (include 6 subtypes)	*POMT1*	10585	chr9	22	AR
		POMT2	29954	chr14	21	
		FKTN	2218	chr9	11	
		FKRP	79147	chr19	1	
		POMGnT1	55624	chr1	24	
		LARGE	9215	chr22	14	
	Muscle-eye-brain disease (MEB)	*POMGnT1*	55624	chr1	24	AR
		FKRP	79147	chr19	1	
		POMT1	10585	chr9	22	
	Fukuyama congenital muscular dystrophy (FCMD) dystrophy-dystroglycanopathy (include 3 subtypes)	*FKTN*	2218	chr9	11	AR

Abbreviations: AD, autosomal dominant; AR, autosomal recessive; XD, X-linked dominant.

Table S3. The numbers of potentially pathogenic mutations identified by the capture sequencing and amplicon sequencing.

Sample ID	Capture Sequencing		Amplicon Sequencing	
	Sequenced	Number of Potentially Pathogenic Mutations	Sequenced	Number of Potentially Pathogenic Mutations
1	YES	0	NO	/
6	YES	0	NO	/
20	YES	0	NO	/
25	YES	0	NO	/
26	YES	0	NO	/
28	YES	0	NO	/
33	YES	0	NO	/
34	YES	0	NO	/
40	YES	0	NO	/
41	YES	0	NO	/
42	YES	0	YES	0
43	YES	0	NO	/
46	YES	0	NO	/
55	YES	0	YES	0
58	YES	1	YES	1
60	YES	0	YES	0
61*	YES	1	NO	/
64	YES	0	YES	0
65	YES	0	YES	0
66	YES	0	YES	0
67	YES	0	YES	0
68	YES	0	YES	0
71	YES	0	YES	0
72	YES	0	YES	0
75	YES	0	YES	0
76	YES	0	YES	0
85	YES	0	NO	/
98	YES	0	YES	0
100	YES	0	NO	/
101	YES	0	YES	0
115*	YES	1	YES	1
120	YES	2	YES	2
124	YES	0	YES	0
125	YES	0	YES	0
126	YES	0	YES	0
128	YES	0	YES	0

Sample ID	Capture Sequencing		Amplicon Sequencing	
	Sequenced	Number of Potentially Pathogenic Mutations	Sequenced	Number of Potentially Pathogenic Mutations
129	YES	0	YES	0
132	YES	0	YES	0
134	YES	0	NO	/
135	YES	2	NO	/
137	YES	1	NO	/
141	YES	1	YES	1
142	YES	0	YES	0
143	YES	1	YES	1
149	YES	0	NO	/
153	YES	0	YES	0
156	YES	0	YES	0
159	YES	0	YES	0
160	YES	0	YES	0
161	YES	0	YES	0
162	YES	2	YES	2
171	YES	0	YES	0
175	YES	1	YES	1
177	YES	0	YES	0
178	YES	0	YES	0
179	YES	1	YES	1
180	YES	0	YES	0
182	YES	0	YES	0
187	YES	1	YES	1
190	YES	1	YES	1
194	YES	1	YES	1
202	YES	2	YES	2
205	YES	0	NO	/
206	YES	0	YES	0
207*	YES	1	YES	1
211	YES	0	YES	0
213	YES	1	YES	1
214	YES	0	NO	/
215	YES	0	NO	/
216	YES	1	NO	/
217	YES	1	YES	1
219	YES	1	NO	/
221	YES	0	YES	0
224	YES	0	NO	/
225	YES	0	YES	0

Sample ID	Capture Sequencing		Amplicon Sequencing	
	Sequenced	Number of Potentially Pathogenic Mutations	Sequenced	Number of Potentially Pathogenic Mutations
227	YES	0	YES	0
228	YES	0	YES	0
231	YES	0	YES	0
232	YES	2	YES	2
238	YES	0	NO	/
239	YES	0	NO	/
241	YES	0	NO	/
247	YES	0	NO	/
252	YES	1	NO	/
255	YES	0	NO	/
256	YES	0	NO	/
257	YES	0	NO	/
260	YES	0	NO	/
261	YES	0	NO	/

* indicates the same pathogenic mutations carried by three patients;

/ indicates no information.

Table S4. The information of the 7 neonates with RTT

Sample ID	Gene	Gender	Genotype	cDNA change	Protein change	Main presentation	Age of onset(days)	Reference(PMID)
61	MECP2	F	Heterozygous	c.602C>T	p.Ala201Val	hypotonia	24	12180070
115	MECP2	F	Heterozygous	c.602C>T	p.Ala201Val	hypotonia	27	12180070
207	MECP2	F	Heterozygous	c.602C>T	p.Ala201Val	hypotonia, difficult feeding	11	12180070
187	MECP2	M	Hemizygous	c.808delC	p.Arg270GlufsX19	hypotonia, difficult feeding	27	10991688
194	MECP2	M	Hemizygous	c.590C>T	p.Thr197Met	hypotonia	18	12180070
137	CDKL5	M	Hemizygous	c.216T>A	p.Ile72=	hypotonia	29	17089071
143	MECP2	F	Heterozygous	c.156C>G	p.His52Gln	hypotonia, difficult feeding, cerebral white matter density decreases	21	
